# Au/CdS Core-Shell Sensitized Actinomorphic Flower-Like ZnO Nanorods for Enhanced Photocatalytic Water Splitting Performance

**DOI:** 10.3390/nano11010233

**Published:** 2021-01-17

**Authors:** Ying Li, Tie Liu, Shuang Feng, Wenshu Yang, Ying Zhu, Yingying Zhao, Zhiyan Liu, Haibin Yang, Wuyou Fu

**Affiliations:** 1 State Key Laboratory of Superhard Materials, Jilin University, Qianjin Street 2699, Changchun 130012, China; yingl18@mails.jlu.edu.cn (Y.L.); zhuying18@mails.jlu.edu.cn (Y.Z.); yingyingz18@mails.jlu.edu.cn (Y.Z.); zhiyanl18@mails.jlu.edu.cn (Z.L.); yanghb@jlu.edu.cn (H.Y.); 2 State Key Laboratory of Luminescence and Applications, Changchun Institute of Optics, Fine Mechanics and Physics, Chinese Academy of Sciences, Changchun 130033, China; liutie@ciomp.ac.cn; 3 College of Physics and Electronic Information, Inner Mongolia University for Nationalities, Tongliao 028000, China; fengshuang@imun.edu.cn; 4 School of Materials Science and Engineering, Jilin University, Changchun 130012, China; yangws16@mails.jlu.edu.cn

**Keywords:** flower-like ZnO nanorods, Au, CdS, photocatalytic water splitting

## Abstract

Herein, a novel actinomorphic flower-like ZnO/Au/CdS nanorods ternary composite photocatalyst is prepared to extend the light-responsive range, reduce the photogenerated charge carriers recombination, and ultimately improve the water splitting performance. Flower-like ZnO nanorods are synthesized by a chemical bath method and the CdS nanoparticles are sensitized by successive ionic layer adsorption and reaction method. Then the Au nanoparticles as co-catalysts are introduced by the photodeposition method to modify the interface of ZnO/CdS for reducing the photogenerated electron recombination rate and further improving the performance of water splitting. Detailed characterizations and measurements are employed to analyse the crystallinity, morphology, composition, and optical properties of the flower-like ZnO/Au/CdS nanorods samples. As a result, the flower-like ZnO/Au/CdS nanorod samples present significantly enhanced water splitting performance with a high gas evolution rate of 502.2 μmol/g/h, which is about 22.5 and 1.5 times higher than that of the pure ZnO sample and ZnO/CdS sample. The results demonstrate that the flower-like ZnO/Au/CdS nanorods ternary composite materials have great application potential in photocatalytic water splitting for the hydrogen evolution field.

## 1. Introduction

Photocatalytic water splitting has attracted considerable research interest as a promising technology for hydrogen generation [1,2]. In order to improve the performance of water splitting, numerous efforts have been devoted to developing semiconductor-based photocatalysis [3,4,5]. As one of the most promising candidates, ZnO with a direct bandgap and low exciton binding energy has been intensively investigated due to its advantages of high electron mobility, low cost, non-toxicity, and simple synthesis process [6,7,8]. The morphology of ZnO plays a crucial role for photocatalytic performance because ZnO is employed as a light harvesting and charge transport material. One-dimensional (1D) nanostructures such as nanowires [9,10,11], nanorods [12,13,14,15] and nanotubes [16], which can provide a direct transport pathway for photogenerated charge, have been widely applied to photocatalytic water splitting. Compared with traditional 1D nanostructures, actinomorphic flower-like nanostructured ZnO exhibits the merits of an efficient charge transport pathway and sufficient surface area simultaneously. Furthermore, the actinomorphic flower-like nanostructure which acts as a light-scattering center can improve the efficiency of light harvesting.

However, two apparent drawbacks hinder the application of ZnO—one limitation is that the large band gap of 3.3 eV limits its light absorption to the ultraviolet region, which is only around 5% of the solar spectrum. Another drawback is the fast recombination rate of photogenerated electrons and holes, leading to a poor photocatalytic performance [17]. Various strategies have been conducted to extend the light responsive range including doping with nonmetallic elements [18,19,20,21], loading with noble metals [22] and sensitizing with narrow band gap semiconductors [23,24,25,26,27,28]. Recently, sensitizing ZnO with narrow band gap semiconductors, including CdS [29], CdSe [30,31] and MoS_2_ [32,33,34], have attracted more and more attention. Among these sensitizer materials, CdS is considered as a promising photocatalyst for water splitting owing to its narrow direct band gap of 2.4 eV and suitable conduction band position for H_2_ evolution. Although the formed ZnO/CdS heterostructure improves the light absorption ability and charge separation efficiency, a considerable portion of the photogenerated electrons are lost at the interface of ZnO/CdS due to the slow electron injection and high recombination rate [35,36]. Therefore, it is highly imperative and significant to reduce the recombination of photogenerated electrons and holes. Noble metal nanoparticles, such as those of Ag [37], Pt [17] and Au [38,39], have been introduced as co-catalysts to increase H_2_ evolution efficiency through reducing the recombination rate. The noble metal nanoparticles inserted between the metal oxide and sensitizer layer play dual roles in improving water splitting performance [40]. On the one hand, the noble metal nanoparticles act as an electron transfer mediator can facilitate the charge transfer at the interface. On the other hand, the surface plasmon resonance of noble metal nanoparticles can improve the light absorption capacity of metal oxide [41,42,43,44,45]. The core-shell structured metal–semiconductor sensitizers were designed and adopted by several researchers to improve the photocatalytic hydrogen evolution efficiency, in which the structure of the ZnO layer consists of nanorods or nanoparticles [13]. To the best of our knowledge, the use of an actinomorphic flower-like ZnO nanorod-based ternary photocatalyst for water splitting has been only rarely reported.

In this study, a novel flower-like ZnO/Au/CdS nanorods ternary composite photocatalyst is prepared by the simple chemical bath method and applied to photocatalytic water splitting. The Au nanoparticles are introduced into the interface of ZnO/CdS forming a ternary heterostructure which improve the electron transport efficiency and light absorption ability simultaneously. In addition, the actinomorphic flower-like ZnO nanorod-based ternary photocatalyst exhibits the advantages of sufficient surface area and favorable electron migration pathway. As a result, the Au/CdS core-shell sensitized flower-like ZnO nanorods sample exhibits a remarkably enhanced gas evolution rate. The detailed characterizations and measurements are conducted to understand the mechanism of the significantly improved photocatalytic water splitting performance.

## 2. Materials and Methods

### 2.1. Materials

Zinc nitrate hexahydrate (Zn(NO_3_)_2_•6H_2_O) and hexamethylenetetramine (HMT) were purchased from Beijing Chemicals Co. Ltd. Cadmium nitrate tetrahydrate (Cd(NO_3_)_2_•4H_2_O) was obtained from Aladdin Chemical reagent Co. Ltd. (Shanghai). The gold chloride hydrate solution (HAuC1_4_•4H_2_O) was purchased from Sinopharm Chemical Reagent Co. Ltd. (Shanghai). Sodium sulfide nonahydrate (Na_2_S•9H_2_O) was purchased from Xilong Chemical Co. Ltd. (Guangdong). The ethanol was obtained from Tianjin Chemical Industry Research Institute. All reagents were used without further purification. Distilled water with a resistivity of 18.0 MΩ cm was used throughout this experiment.

### 2.2. Preparation of Actinomorphic Flower-Like ZnO Nanorods

The actinomorphic flower-like ZnO nanorods were prepared through the chemical bath method. Typically, a 100 mL aqueous solution of 0.1 M Zn(NO_3_)_2_•6H_2_O and 100 mL aqueous solution of 0.1 HMT were mixed together and kept under mild magnetic stirring for 5 min. Then, the mixture solution was transferred into a 500 mL flask and heated at 90 °C for 24 h and subsequently cooled down to room temperature naturally. Finally, the obtained white products were centrifuged and washed with deionized water and ethanol, dried at 60 °C in air, and annealed at 550 °C for 2 h.

### 2.3. Preparation of Au/CdS Core-Shell Sensitized Flower-Like ZnO Nanorods

Au nanoparticles were deposited on the surface of ZnO by the photodeposition method. First, 100 μL aqueous solution of 0.1 M HAuC1_4_•4H_2_O was added to a beaker containing 100 mL deionized water and stirred for 5 min. Then, 500 mg ZnO power was added into the Au precursor solution and stirred for 5 min. Under stirring conditions, the mixed solution was irradiated with a 300 W high pressure mercury lamp for 5 min. The obtained samples were centrifuged and washed and then dried in an oven at 80 °C for 12 h.

The deposition of CdS was conducted by a successive ionic layer adsorption and reaction method. In a typical procedure, the samples were dipped in 0.5 M Cd(NO_3_)_2_•4H_2_O alcohol solution and stirred for five minutes and then the samples were collected and washed with alcohol. After that, the samples were then immersed in an 0.5 M Na_2_S•9H_2_O aqueous solution and stirred for five minutes and the obtained products were centrifuged and washed. This process was repeated five times and eventually the samples were annealed at 300 °C for 1 h in air atmosphere.

### 2.4. Characterization

The crystal structure of the samples was characterized by a Rigaku D/max-2500 X-ray diffractometer with Cu Kα (λ = 1.5418 Å) radiation. The morphology and microstructure of the samples were observed by FEI MAGELLAN 400 field-emission scanning electron microscopy (FESEM). The elemental analysis was conducted using energy dispersive X-ray spectrometry (EDS) and EDS mapping on the FESEM. Transmission electron microscopy (TEM) and high-resolution transmission electron microscopy (HRTEM) were characterized by a JEM-2100F transmission electron microscope operating at 200 kV. X-ray photoemission spectroscopy (XPS) spectra were obtained with a PHI 5200 mode XPS system using an Al Ka X-ray source. The UV–vis diffused reflectance absorption spectra were obtained using a UV-3150 double-beam spectrophotometer. The photoluminescence (PL) spectra were recorded using a F-7000 Fluorescence Luminescence Spectrophotometer with an excitation wavelength of 325 nm. The transient photocurrent response measurement was performed in a standard three-electrode configuration, which is made of a quartz cell and linked with the electrochemical workstation (CH Instruments, model CHI601 C). The samples deposited on Fluorine-doped tin oxide glass (FTO) were used as the working electrode, a platinum net as the counter electrode, and a saturated Ag/AgCl electrode as the reference electrode. The electrolyte was a mixture aqueous solution of 0.25 M Na_2_S•9H_2_O and 0.35 M Na_2_SO_3_.

### 2.5. Photocatalytic Hydrogen Evolution

Photocatalytic activity for water splitting is carried out in a closed Pyrex reactor connected to a closed glass gas collection system. The reactor was filled with a mixture aqueous solution of 0.25 M Na_2_S•9H_2_O and 0.35 M Na_2_SO_3_ which served as a sacrificial agent and 100 mg of photocatalyst samples were dispersed in 400 mL of the solution and stirred during the photoreaction. A 500 W xenon lamp (AM 1.5G) at an irradiance of 100 mWcm^−2^ was used as a light source for photocatalytic studies. The schematic diagram of experimental arrangement is shown in Figure S1.

## 3. Results

**Figure 1:**
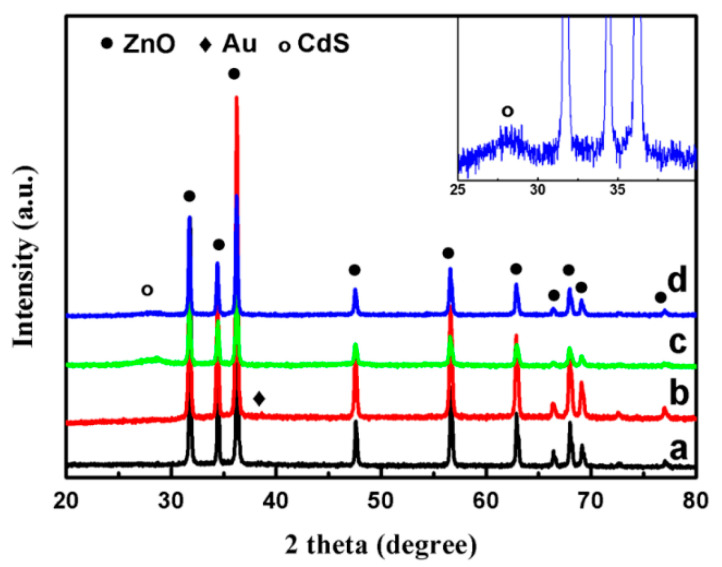
XRD patterns of (**a**) ZnO; (**b**) ZnO/Au; (**c**) ZnO/CdS; and (**d**) ZnO/Au/CdS samples. Inset: magnification images of a portion of the XRD pattern.

**Figure 2:**
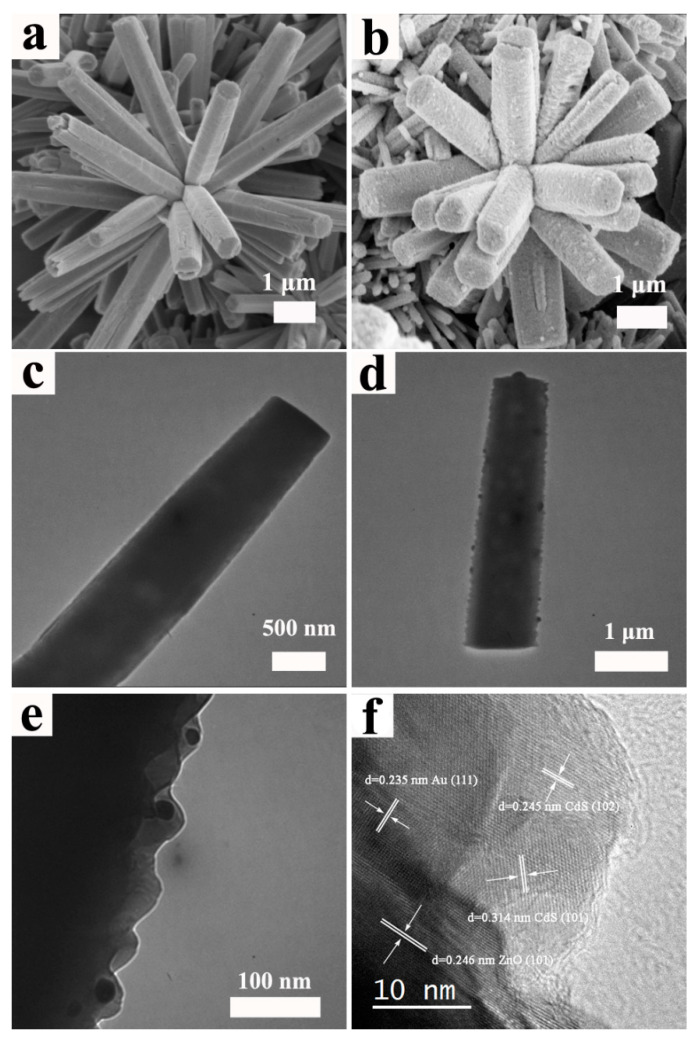
FESEM images of (**a**) ZnO sample and (**b**) ZnO/Au/CdS sample; TEM images of (**c**) ZnO sample and (**d**,**e**) ZnO/Au/CdS sample; (**f**) HRTEM image of ZnO/Au/CdS sample.

To identify the effects of Au/CdS core-shell sensitization on the crystal structure of ZnO, XRD patterns of ZnO, ZnO/Au, ZnO/CdS and ZnO/Au/CdS samples are measured. As shown in Figure 1, the XRD diffraction peaks at 31.80°, 34.45° and 36.28° correspond well with the (100), (002) and (101) planes of hexagonal wurtzite ZnO [46]. No diffraction peaks from any other impurities are detected which indicates high purity and crystallinity of ZnO sample. The ZnO/Au sample exhibits a new diffraction peak at 38.28°, corresponding to (111) plane of Au nanoparticles [JCPDS 1-1172] [47,48], which indicates the successful decoration of Au nanoparticles on flower-like ZnO nanorods. The peak of Au nanoparticles is extremely weak, which is attributed to the relatively low decoration amount. The ZnO/CdS and ZnO/Au/CdS samples show a fresh diffraction peak at 28.38°, which can be indexed to the (101) plane of CdS [JCPDS 1-780]. No apparent peaks of Au can be observed in the ZnO/Au/CdS sample, which is attributed to low decoration amount and the subsequent coating of CdS. The above results are consistent with the HRTEM results in Figure 2f. In addition, it can be found that the intensities of the ZnO diffraction peaks are reduced after the deposition of CdS, which means that the flower-like ZnO nanorods are fully covered by CdS nanoparticles.

The surface morphologies of ZnO and ZnO/Au/CdS sample are investigated by FESEM. As shown in Figure 2a, the ZnO sample shows an actinomorphic flower-like microstructure assembled by the closely packed nanorods. It can be seen that the nanorods have a smooth surface, the diameter of the nanorod is about 500 nm and the length of nanorods is 5 μm approximately. Figure 2b shows the surface morphology of the ZnO/Au/CdS sample. Some nanoparticles are clearly observed on the surface of ZnO nanorods, indicating that Au/CdS core-shell nanoparticles have been successfully deposited on the surface of the actinomorphic flower-like ZnO nanorods. The detailed microstructure is further investigated by TEM and HRTEM. Figure 2c shows the TEM image of a bare ZnO nanorod, in which it can be seen that the surface of a bare ZnO nanorod is smooth. Figure 2d shows the TEM image of ZnO/Au/CdS sample. It can be seen that Au/CdS core-shell nanoparticles are loaded on the surface of the ZnO nanorod. In addition, as seen from the TEM image, the Au/CdS core-shell nanoparticles are uniformly distributed on the surface of ZnO nanorod. As shown in Figure 2e, the Au nanoparticles and CdS nanoparticles are clearly observed and the Au nanoparticles are located between the ZnO and CdS, which further proves that the form of the Au/CdS core-shell structure and the Au/CdS core-shell nanoparticles correspond with ZnO nanorods. Specifically, the Au core has an average diameter of 20 nm and the CdS shell has an average thickness of 30 nm. Figure 2f shows the HRTEM image of ZnO/Au/CdS heterojunction and the distinct lattice fringes of the sample demonstrate the highly crystalline nature of ZnO/Au/CdS ternary composite semiconductor material. The measured lattice fringe spacings in Figure 2f are consistent with the d-spacings of ZnO, Au, and CdS. The lattice fringe spacing d(101) = 0.246 nm corresponds to hexagonal wurtzite ZnO [JCPGS 79-0205], the lattice spacing d(101) = 0.314 nm and d(102) = 0.245 nm of the nanocrystalline on the right of HRTEM image correspond to CdS [JCPDS 1-780] and the lattice fringe spacing d(111) = 0.235 nm corresponds to Au [JCPDS 1-1172]. Meanwhile, the lattice interlacing occurs at the interface of ZnO, Au, and CdS by certain angles which indicates the growth relationship of the ternary composite semiconductor material rather than a simple physical adsorption. This type of interface is conducive to electron transport and a reduction in carrier recombination loss.

**Figure 3:**
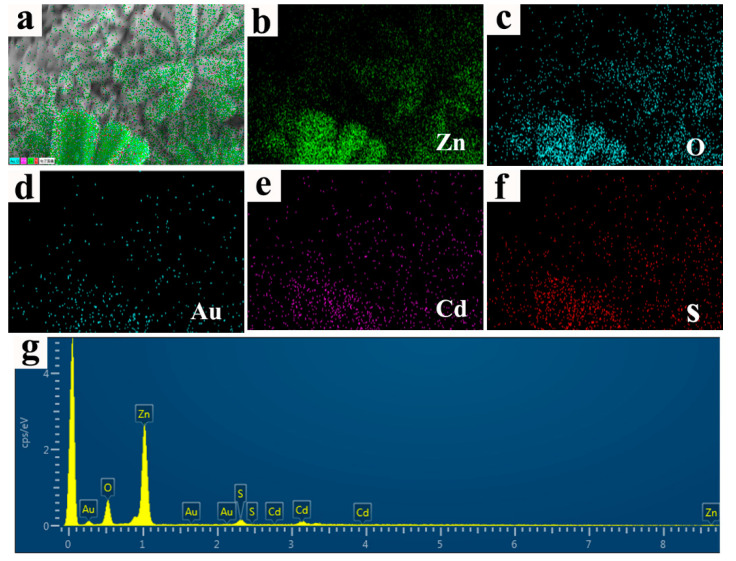
(**a**–**f**) EDS mapping images of the ZnO/Au/CdS sample; and (**g**) EDS image of the ZnO/Au/CdS sample.

The composition and elemental distribution of the ZnO/Au/CdS sample are investigated by EDS and EDS mapping. The EDS mapping image of ZnO/Au/CdS sample are shown in Figure 3a–f. It can be seen that Zn, O, Au, Cd, and S elements are homogenously distributed on the entire surface of actinomorphic flower-like ZnO nanorods sample, suggesting that the Au/CdS core-shell structures are grown homogeneously on the surface of the sample. The typical EDS image shown in Figure 3g exhibits that the ZnO/Au/CdS sample is composed of Zn, O, Au, Cd, and S elements.

**Figure 4:**
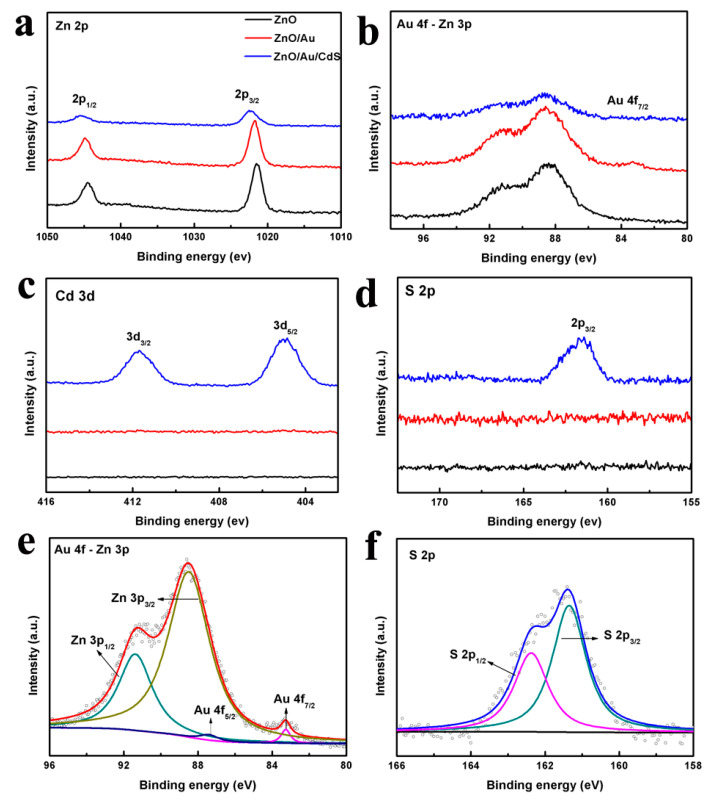
(**a**) Zn 2p, (**b**) Au 4f-Zn 3p, (**c**) Cd 3d, and (**d**) S 2p peaks in XPS spectra of ZnO, ZnO/Au and ZnO/Au/CdS samples, respectively; (**e**) Au 4f-Zn 3p peaks of ZnO/Au sample and (**f**) S 2p peaks of ZnO/Au/CdS sample.

The XPS spectra are analyzed to further explore the surface elemental composition. Figure 4 shows a comparison of the Zn 2p, Au 4f-Zn 3p, Cd 3d and S 2p XPS spectra of ZnO, ZnO/Au and ZnO/Au/CdS samples, respectively. As presented in Figure 4a, the two peaks at 1044.6 and 1021.6 eV could be assigned to the Zn 2p1/2 and Zn 2p3/2 peaks of ZnO [39,49]. The intensity of the Zn 2p peaks in the ZnO/Au/CdS sample is lower than that of ZnO and ZnO/Au, which is attributed to the coating of CdS. Moreover, the Zn 2p peaks exhibit a small shift after the sequential deposition of Au and CdS, which is attributed to the electron transfer in the composite materials [50]. As shown in Figure 4b, the Au 4f7/2 peak signal is observed in the ZnO/Au sample but almost disappeared in the ZnO/Au/CdS sample, which is attributed to the simultaneous effect of CdS shell coating and low decoration amounts. To further analyze the decoration of Au, the Au 4f-Zn 3p curve of ZnO/Au sample is fitted, as shown in Figure 4e. The peaks observed at 91.4 and 88.5 eV are ascribed to Zn 3p1/2 and Zn 3p3/2, respectively [49]. The binding energies of Au 4f5/2 and Au 4f7/2 at 87.4 and 83.3 eV are attributed to the metallic gold Au^0^ [39,47,49,50,51]. A relative negative shift of 0.7 eV of the Au 4f7/2 peak compared to bulk Au (Au 4f7/2 peak at 84.0 eV) is observed, which is ascribed to the electron transfer from oxygen vacancies of ZnO to Au, leading to a lower binding energy of Au 4f7/2 in ZnO/Au sample [39,50,51]. The Cd 3d peaks (Cd 3d3/2 and Cd 3d5/2 at 411.6 eV and 404.9 eV, respectively) and S 2p peaks (S 2p1/2 and S 2p3/2 at 162.4 eV and 161.2 eV, respectively) are observed in ZnO/Au/CdS sample, and the binding energy values of these peaks are consistent with the binding energies of CdS [47,48]. All the results provide strong evidence for the successful coating of Au and CdS nanoparticles on the surface of flower-like ZnO nanorods.

**Figure 5:**
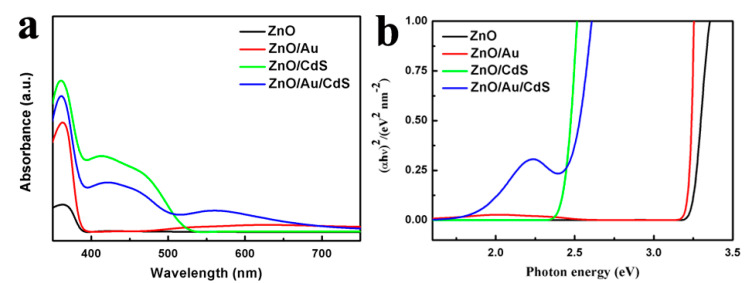
(**a**) UV-vis absorption spectra and (**b**) (*ahv*)^2^-photon energy curves of the ZnO, ZnO/Au, ZnO/CdS, and ZnO/Au/CdS samples.

The optical absorption properties of the samples are investigated by UV–vis absorption spectra. As shown in Figure 5a, the light absorption range of bare ZnO sample is limited to the ultraviolet region due to its wide optical band gap with an absorption edge of about 380 nm. As Au nanoparticles are attached on ZnO nanorods, the ZnO/Au sample shows a broad peak at 560 nm which is caused by the surface plasmon resonance absorption of Au nanoparticles [35]. For the ZnO/CdS sample, the light absorption edge is red-shifted to 530 nm and the light absorption intensity is significantly enhanced. This indicates that the sensitization of CdS can effectively improve the utilization of visible light. Compared with the ZnO/CdS sample, the ZnO/Au/CdS sample exhibits an extended photoresponse range. Surface plasmon resonance absorption of Au nanoparticles is also found in the ZnO/Au/CdS sample, which also shows a CdS absorption band at 530 nm. The band gap energy (E_g_) is calculated according to Equation (1), where *a* is the absorption coefficient, *h* is Planck’s constant, *v* is the frequency, and c is a constant, respectively [52].

(*αhv*)^2^ = *c* (*hv* − E_g_) (1)

The E_g_ values of samples can be obtained by extrapolating the linear portion of plots at *a* = 0. As shown in Figure 5b, the calculated band gap energy of ZnO, ZnO/CdS and ZnO/Au/CdS samples are 3.25, 2.40, and 1.75 eV, respectively. The improved visible light absorption ability makes this type of ZnO/Au/CdS ternary composite semiconductor material a promising candidate for applications in the photocatalytic water splitting field.

**Figure 6:**
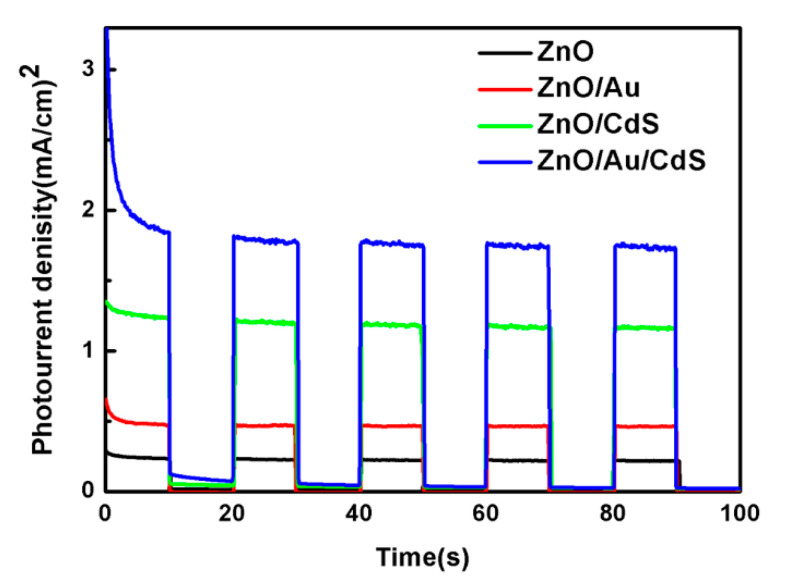
Transient photocurrent response of ZnO, ZnO/Au, ZnO/CdS and ZnO/Au/CdS sample.

To further study the separation and transport behavior of photogenerated charge carriers, the samples are coated on FTO substrates and the transient photocurrent response is investigated. Figure 6 presents the photocurrent response of the samples under AM 1.5G illumination. In all of the cases, the photocurrent density increases rapidly to a stable state value when the light is on, and the photocurrent density drops to a negligible value when the light is off. The results demonstrate the efficient electron transport performance in actinomorphic flower-like ZnO nanorods. The bare actinomorphic flower-like ZnO nanorod sample shows the lowest photocurrent density, which is attributed to the large band gap and quick recombination of photogenerated charge carriers. The photocurrent response of ZnO/Au sample is a little higher than that of bare ZnO. The ZnO/CdS sample exhibits obviously improved photocurrent intensity, which implies that the sensitization of CdS nanoparticles increases the utilization of visible light. Compared to the ZnO/CdS sample, the ZnO/Au/CdS sample exhibits higher photocurrent density, which can be ascribed to the improved separation and transport behavior of photogenerated charge carriers due to the modification of Au nanoparticles. The results clearly demonstrate that decoration with Au nanoparticles reduces the interface recombination rate and improves the charge transfer performance.

**Figure 7:**
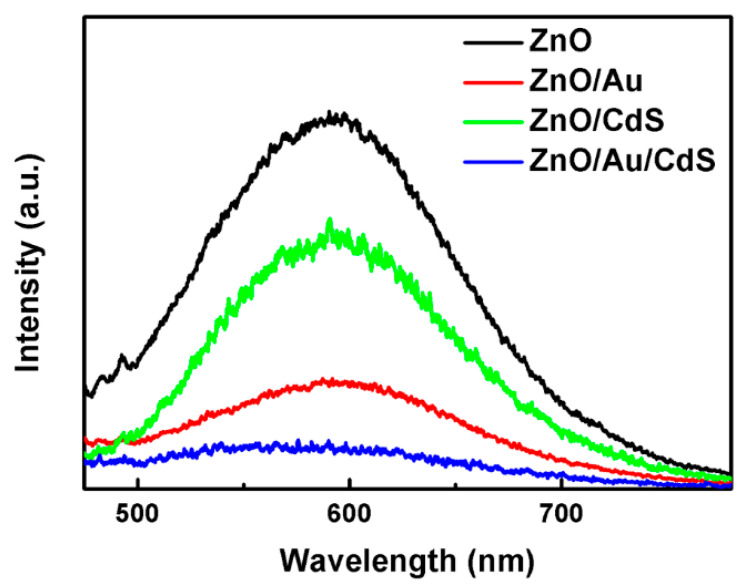
Photoluminescence (PL) spectra of ZnO, ZnO/Au, ZnO/CdS and ZnO/Au/CdS samples.

To further clarify the effect of Au nanoparticles on photogenerated charge carrier separation and transfer, PL spectra of the samples are analyzed. Figure 7 shows the PL spectra of the ZnO, ZnO/Au, ZnO/CdS and ZnO/Au/CdS samples, which are deposited on FTO substrates. It can be observed that the ZnO sample exhibits the highest PL intensity, suggesting the serious recombination of photogenerated electrons and holes in the photocatalyst. The PL intensity of the ZnO/CdS sample decreased to some extent due to the formation of the energy level gradient at the interface of ZnO/CdS, which benefits the spatial separation and transfer of photogenerated charge carriers [49]. After the loading of Au nanoparticles, the PL intensities of ZnO/Au and ZnO/Au/CdS samples are dramatically reduced, indicating that the Au nanoparticles play an important role in the efficient suppression of the recombination of photogenerated electrons and holes [17,48,53]. The obviously improved separation and transfer efficiency of photogenerated charge carriers in ZnO/Au/CdS sample makes this type of ternary composite photocatalyst more promising for applications in water splitting.

**Figure 8:**
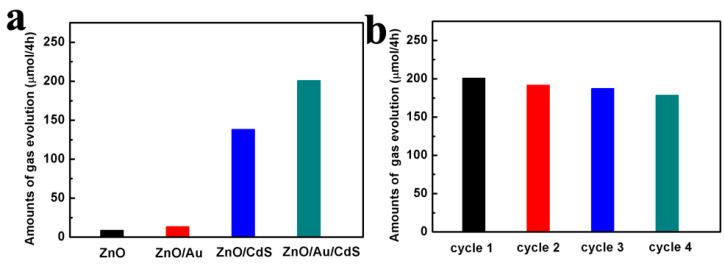
(**a**) The amounts of gas evolution of the ZnO, ZnO/Au, ZnO/CdS and ZnO/Au/CdS samples (100 mg of catalyst for 4 h); (**b**) the cycling stability of the ZnO/Au/CdS photocatalyst (4 h as a cycle).

The ZnO, ZnO/Au, ZnO/CdS and ZnO/Au/CdS samples are used for photocatalytic water splitting for H_2_ evolution. As shown in Figure 8a, both ZnO and ZnO/Au samples present low gas evolution rates of 22.3 and 33.5 μmol/g/h, respectively. The ZnO/CdS sample shows a much higher gas evolution rate of 346.0 μmol/g/h, which can be ascribed to the formation of type Ⅱ heterojunction structure and the extension of light absorption range. After the introduction of Au nanoparticles into the ZnO/CdS system, the ZnO/Au/CdS ternary composite photocatalyst sample shows a remarkably improved photocatalytic performance with a gas evolution rate of 502.2 μmol/g/h, which is about 1.5 times than that of ZnO/CdS sample. The results suggest that the Au and CdS nanoparticles have a synergistic effect for the improvement of photocatalytic water splitting. Meanwhile, in order to prove the stability of the ZnO/Au/CdS ternary composite photocatalyst, the cycling stability measurement is conducted. As shown in Figure 8b, the amounts of gas evolution maintain for four cycles with slight decay and the slight decrease in photocatalytic activity is attributed to the ZnO photocorrosion phenomena [23,53]. The above results indicate that the actinomorphic flower-like ZnO/Au/CdS ternary composite material is a promising photocatalyst for water splitting.

**Figure 9:**
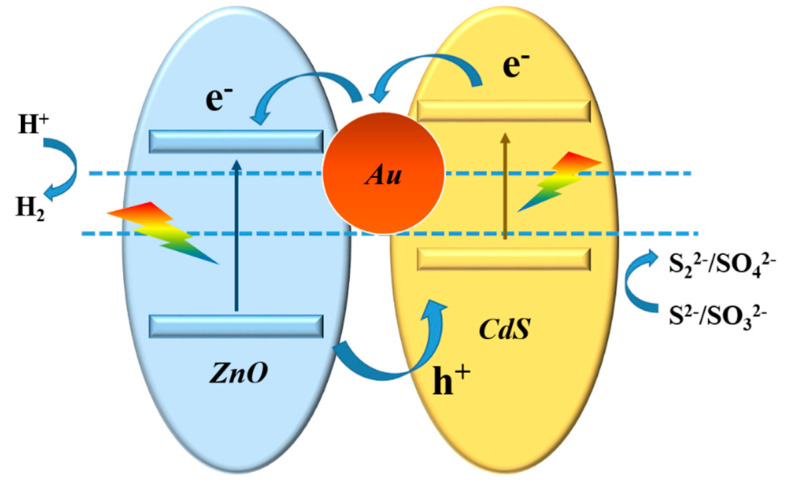
Schematic illustration of charge transfer process and gas evolution mechanism of ZnO/Au/CdS sample.

The mechanism of photocatalytic water splitting for actinomorphic flower-like ZnO/Au/CdS nanorods ternary composite photocatalyst is shown in Figure 9. The photogenerated electrons are excited in the ZnO/Au/CdS ternary composite photocatalyst sample under the light irradiation, and the photogenerated electrons in the conduction band of CdS are transferred to the conduction band of ZnO via Au nanoparticles, while the photogenerated holes are accumulated at the valence band of CdS to keep the effectively spatially separation of electrons and holes. Then, the gas is produced via the reduction reaction of H^+^ by the photogenerated electrons at the surface of ZnO and the photogenerated holes react with an electron donor at the surface of CdS. Although the hydrogen evolution can be achieved with the traditional ZnO/CdS heterojunction structure, the major shortcoming is the slow electron transport process. The processes of exciton separation are very fast, and the internal electron transfer is highly accelerated by the modification of Au nanoparticles. The accelerated electron transfer in the ZnO/Au/CdS ternary composite photocatalyst results in the higher gas generation rate than that of the ZnO/CdS sample. Thus, several reasons are proposed here for the enhanced photocatalytic water splitting performance of the actinomorphic flower-like ZnO/Au/CdS nanorod ternary composite photocatalyst. First, the actinomorphic flower-like ZnO nanorod structure provides sufficient surface area for sensitizer deposition and sacrificial agent penetration, and provides the direct charge transport pathway simultaneously. Second, the light absorption performance is remarkably improved via the modification of the Au/CdS core-shell structure. Last, the introduction of Au nanoparticles increases the separation and transfer efficiency of photogenerated charge carriers.

## 4. Conclusions

In summary, the ZnO/Au/CdS ternary composite photocatalyst based on actinomorphic flower-like nanorods is successfully fabricated. The flower-like nanorod-structured photocatalyst enhances the water splitting performance due to the advantages of sufficient surface area and direct electron transfer pathway. The introduction of Au nanoparticles between ZnO and CdS further increases the gas evolution rate, which is attributed to the electron transfer mediator function of Au nanoparticles. The actinomorphic flower-like ZnO/Au/CdS nanorod sample exhibits the highest gas evolution rate of 502.2 μmol/g/h. Overall, the present work provides an insight for preparing highly efficient heterostructure photocatalysts by exploiting the synergistic effects of a flower-like nanorod structure and Au nanoparticles.

## Data Availability

The data is available on request from the corresponding author.

## Supplementary Materials

The following are available online at https://www.mdpi.com/2079-4991/11/1/233/s1. Figure S1: schematic diagram of experimental arrangement for measurement.
