# Supplementary material for: Au/CdS Core-Shell Sensitized Actinomorphic Flower-Like ZnO Nanorods for Enhanced Photocatalytic Water Splitting Performance
Source: Nanomaterials (Basel). 2021 Jan 17;11(1):233. doi: 10.3390/nano11010233 (PMC7830535; doi:10.3390/nano11010233)
Supplement: Supplementary file 1 [file nanomaterials-11-00233-s001.pdf]

Supporting Material

Au/CdS core-shell sensitized actinomorphic flower-like ZnO nanorods for enhanced photocatalytic water splitting performance

Ying Li [1], Tie Liu [2],Shuang Feng [3], Wenshu Yang [4], Ying Zhu [1], Yingying Zhao [1],Zhiyan Liu [1], Haibin Yang [1] and Wuyou Fu[1,*]

[1] State Key Laboratory of Superhard Materials, Jilin University, Qianjin Street 2699, Changchun, 130012, P. R. China; yingl18@mails.jlu.edu.cn; (Y.L.); zhuying18@mails@jlu.edu.cn; (Y. Z.); yingyingz18@mails.jlu.edu.cn; (Y.Z); zhiyanl18@mails.jlu.edu.cn; (Z.L.); yanghb@jlu.edu.cn; (H.Y.);
[2] State Key Laboratory of Luminescence and Applications, Changchun Institute of Optics, Fine Mechanics and Physics, Chinese Academy of Sciences, Changchun, 130033, P. R. China; liutie@ciomp.ac.cn; (T.L.);
[3] College of Physics and Electronic Information, Inner Mongolia University for Nationalities, Tongliao 028000, P. R. China; fengshuang@imun.edu.cn; (S.F.);
[4] School of Materials Science and Engineering，Jilin University, Changchun, 130012, P. R. China; yangws16@mails.jlu.edu.cn; (W.Y.);
* Correspondence: fuwy@jlu.edu.cn; (W.F.); Tel.: +86-431-8516-8763-801; Fax: +86-431-8516-8763-801

Citation: Li, Y.; Liu, T.; Feng, S.; Yang, W.; Zhu, Y.; Zhao, Y.; Liu, Z.; Yang, H.; Fu, W. Au/CdS Core-Shell Sensitized Actinomorphic Flower-Like ZnO Nanorods for Enhanced Photocatalytic Water Splitting Performance. *Nanomaterials* **2021**, *11*, 233. https://doi.org/10.3390/nano11010233

Received: 26 December 2020
Accepted: 14 January 2021
Published: 17 January 2021

Publisher's Note: MDPI stays neutral with regard to jurisdictional claims in published maps and institutional affiliations.

Copyright: © 2021 by the authors. Submitted for possible open access publication under the terms and conditions of the Creative Commons Attribution (CC BY) license (http://creativecommons.org/licenses/by/4.0/).

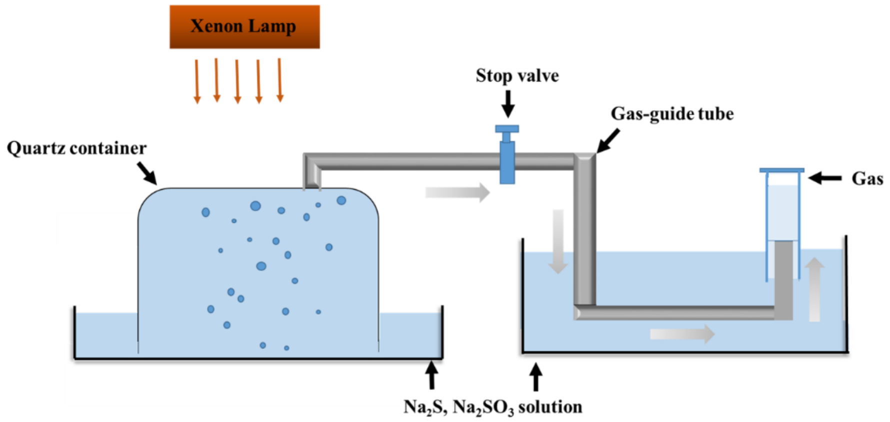

Figure S1. Schematic diagram of experimental arrangement for measurement.

Figure S1 shows the schematic diagram of experimental arrangement for photocatalytic water splitting measurement [1].

[1] Yang, W.; Zhu, G.; Wang, J.; Feng, S.; Yang, J.; Su, P.; Fu, W.; Yang, H. Preparation of NiO by simple heat treatment method and its application in photocatalytic water splitting. *Catal. Lett.* **2019,** *149*, 1680–1689.
